# Synergistic Influence of Multi-Walled Carbon Nanotubes and Nanosilica Powder on Mechanical Performance of Mortar with Demolished Concrete Waste Aggregate and Polypropylene Fibers Addition Using Taguchi Design of Experiment

**DOI:** 10.3390/ma18245485

**Published:** 2025-12-05

**Authors:** Daniel Lepadatu, Loredana Emanuela Judele, Dana Roxana Bucur, Isabela Maria Simion, Ioana Sorina Entuc, Eduard Proaspat, Razvan Ionut Teodorescu, Abdessamad Kobi, Santiago Garcia-Granda

**Affiliations:** 1 Civil Engineering Department, Faculty of Civil Engineering and Buildings Services, Technical University Gheorghe Asachi from Iasi, 700050 Iasi, Romaniaioana-sorina.entuc@academic.tuiasi.ro (I.S.E.); 2 Transportation Infrastructure Engineering Department, Faculty of Architecture and Urban Planning, Technical University of Moldova, 2028 Chisinau, Moldova; eduard.proaspat@iit.utm.md; 3 Concrete Structures, Building Materials, Technology, and Management Department, Faculty of Civil Engineering, Technical University Gheorghe Asachi from Iasi, 700050 Iasi, Romania; 4 Department of Control, Expertise and Services, Faculty of Food and Animal Sciences, “Ion Ionescu de la Brad” Iasi, University of Life Sciences, 8 Mihail Sadoveanu Alley, 700490 Iasi, Romania; 5 Department of Pedotechnics, Faculty of Agriculture, “Ion Ionescu de la Brad” Iasi, University of Life Sciences, 700490 Iasi, Romania; isabela.simion@iuls.ro; 6 Faculty of Land Reclamation and Environmental Engineering, University of Agronomic Sciences and Veterinary Medicine Bucharest, 011464 Bucharest, Romania; razvan.teodorescu@usamv.ro; 7 Angevin Laboratory for Systems Engineering Research Polytech Angers—LARIS, University of Angers, 49000 Angers, France; 8 Department of Physical and Analytical Chemistry, Faculty of Chemistry, University of Oviedo, 33006 Oviedo, Spain

**Keywords:** recycled concrete, carbon nanofibers, nanosilica, concrete waste aggregate, mechanical performances, Taguchi design of experiments

## Abstract

This study investigates the synergistic influence of multi-walled carbon nanotubes (MWC-NTs), nanosilica powder (NSP), and polypropylene fiber waste (PFW) on the mechanical performance of mortar incorporating demolished concrete waste aggregates (DCWA). The replacement of natural aggregates with DCWA typically results in strength reductions and weak interfacial transition zones; therefore, the combined use of nanomaterials and microfibers is proposed as a mitigation strategy. A Taguchi Design of Experiments (DOE) approach was employed to optimize mix parameters, including MWCNT dosage, NSP content, PFW volume fraction, and DCWA replacement level. Mortar mixtures were prepared with MWCNTs (0–0.1% by binder weight), NSP (0–2% by binder weight), PFW (0–0.3% by volume), and DCWA (0–20% replacement of fine sand). Mechanical performance was assessed through compressive and flexural strength tests. A combined statistical approach using the Pareto chart and ANOVA identified the most influential parameters and their respective contributions to the response variable. The innovative aspect of this research lies in the synergistic integration of MWCNTs, NSP, demolished concrete waste, and polypropylene fiber waste within the mortar matrix, with the incorporation of nanomaterials specifically intended to compensate for the strength reduction typically induced by the use of demolition concrete waste aggregates. Although a potential nano-scale synergy between MWCNTs and NSP was initially considered, the experimental results indicated that the most relevant synergistic effects occurred among broader mix parameters rather than specifically between the two nanomaterials. Even so, when assessed individually, both nanomaterials contributed to improving the mechanical characteristics of the mortar—particularly nanosilica, which demonstrated a more pronounced effect—yet these individual enhancements did not translate into a distinct synergistic interaction between MWCNTs and NSP. The Taguchi DOE proved to be an efficient tool for multiple factor analysis, enabling reliable identification of the most influential parameters with a minimum number of tests. Its application facilitated the development of mortar mixtures that effectively integrate demolition waste while achieving enhanced mechanical performance through nano- and micro-scale reinforcement.

## 1. Introduction

The rapid growth of construction and demolition activities has resulted in a significant increase in demolition waste, thereby raising concerns about sustainable resource management and environmental impact. One promising approach to addressing these issues is the utilization of recycled demolished concrete waste aggregates in concrete production [1,2,3,4,5,6]. While this practice contributes to waste reduction and resource conservation, it is often associated with a decrease in mechanical performance and durability compared to conventional concrete, thus requiring innovative modification strategies.

Nanomaterials have emerged as highly effective modifiers for cementitious composites due to their unique surface properties and ability to alter the microstructure at the nanoscale [7,8,9,10,11,12,13,14,15]. Multi-walled carbon nanotubes (MWCNTs) are particularly effective in bridging microcracks and enhancing load transfer across the cement matrix [16,17], while nanosilica particles contribute to densification of the microstructure through the pozzolanic activity and pore refinement. Simultaneously, polypropylene fibers enhance ductility, crack resistance, and post-cracking behavior, further improving the overall performance of the composite.

Although several studies have investigated the incorporation of individual nanomaterials in cementitious systems, research combining different types of nanoparticles [18,19] is still in its early stages. The wide variety of available nanomaterials [16,17,18,19], each with distinct physicochemical properties, opens up numerous possibilities for synergistic interactions that, could further enhance the performance of cement-based composites. However, the optimization of such multi-nanomaterial systems remains a significant challenge, as factors such as particle compatibility, dispersion stability, dosage, and interaction with the cement hydration process can greatly influence the final properties of the composite. Therefore, further experimental and analytical studies are needed to identify the most effective nanoparticle combinations and to establish reliable guidelines for their use in sustainable and high-performance cementitious materials.

Numerous researchers have explored various combinations of different types of nanoparticles [16,17,18,19,20,21,22,23] with the aim of optimizing their dosage for improved mechanical performance, these works have generally not taken into account other crucial parameters such as the water-to-cement ratio, cement content, aggregate characteristics, and the use of various admixtures and additives. Neglecting these factors can significantly influence the observed results, and therefore, the reported improvements in mechanical properties may not fully reflect the potential performance of the nanomodified cementitious systems under more controlled and comprehensive experimental conditions.

In most studies conducted, researchers have reported a more pronounced favorable influence of nanosilica [6,8,9,14,15,20,21] on the mechanical and microstructural properties of cementitious composites. This effect is primarily attributed to its high specific surface area, enhanced pozzolanic reactivity, and its ability to generate additional calcium silicate hydrate (C–S–H) phases, leading to microstructural densification and reduced porosity. However, the optimization of nanosilica dosage remains a challenging task, considering the multitude of parameters that can affect this complex process—including particle characteristics and dispersion efficiency, the water-to-cement ratio, and potential synergistic interactions among constituents. Consequently, identifying the optimal proportions and the most suitable experimental conditions remains a highly active and promising research direction.

The incorporation of CNTs into cementitious materials [16,17,19,20,21,22,23,24] significantly enhances mechanical strengths due to their ability to efficiently transfer stresses and inhibit microcrack propagation within the matrix. In specific configurations and optimal dosages, the combined use of MWCNTs [16,17] with other nanostructures [19,20,21,23,25], such as NSP [6,8,9,25], can further amplify these effects by synergistically influencing interfacial mechanisms and the deformation behavior of the composite. Consequently, CNTs and their combinations with complementary nanomaterials not only improve mechanical performance but can also fundamentally alter the response of cement-based materials [26] under complex loading conditions.

The Taguchi Design of Experiments (DOE) methodology [27,28,29] provides a robust statistical framework to systematically investigate the influence of these materials and their interactions. This approach [27,28,29,30] enables the identification of optimal parameter settings with minimal experimental effort, thereby enhancing both efficiency and reliability in experimental design.

The novelty of this research lies in the integrated use of MWCNTs, nanosilica, demolition waste aggregates, and polypropylene fibers within the mortar matrix, with the nanomaterials incorporated to mitigate the strength loss typically associated with recycled aggregates. While their combined action was initially expected to yield a direct nano-scale synergy, the experimental findings showed that the most relevant interactions occurred at the broader mix-parameter level rather than specifically between the two nanomaterials.

While numerous studies have separately examined the effects of nanomaterials [16,17,19,25], recycled aggregates [13,14,15,23,25,26] or fiber reinforcement, the combined influence of these four components remains largely unexplored in the literature. The investigation of their joint effect offers the potential to establish a new pathway for producing sustainable, high-performance cementitious materials, thereby addressing both environmental and structural performance challenges.

In this study, the experimental design is implemented using the Taguchi method, which provides a modern and efficient approach suitable for such a complex investigation. This method highlights the individual influence of each factor and also allows the evaluation of additional parameters beyond the two types of nanoparticles, such as water-to-cement ratio, cement content, aggregate characteristics, and admixtures. As a result, the study adopts an original approach, offering a more comprehensive understanding of the interactions between all components and enabling a detailed optimization of the mortar performance.

## 2. Materials and Methods

### 2.1. Introduction

The cement employed in the preparation of the mortar samples was Portland cement (CEM I 42.5 class N).sourced from Holcim [31] România. Its main physical properties, together with those of the fine aggregates, are presented in Table 1. These characteristics meet standard requirements, confirming the suitability of the materials for mortar production and their ability to ensure adequate workability and strength development.

### 2.2. Polygranular Sand Aggregate

**Figure 1:**
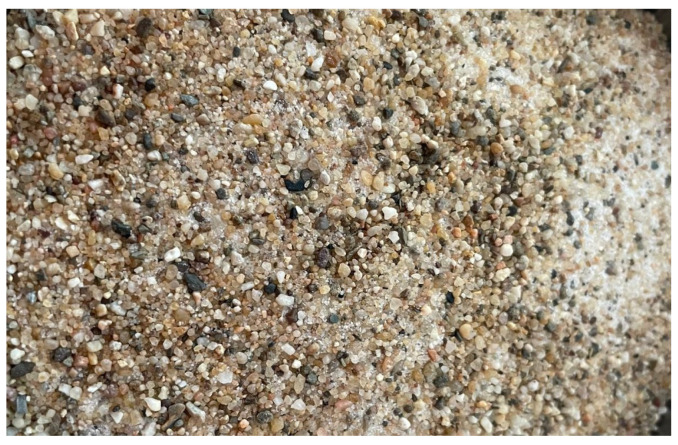
Polygranular sand.

The aggregate used for the preparation of plastic mortar specimens is a standardized polygranular sand (Figure 1), in accordance with the specifications of the European standard EN 196-1 [32]. It consists of four distinct granulometric fractions, each characterized by specific particle sizes and morphological features, as presented in Table 2. The use of such a standardized aggregate aims to eliminate variability associated with natural materials, thereby ensuring controlled experimental conditions and reproducible results. The standardization of granulometry is a key factor in enabling comparability of data across laboratories and in providing a reliable assessment of cement-based material performance.

### 2.3. Polipropylene Fibers Waste

**Figure 2:**
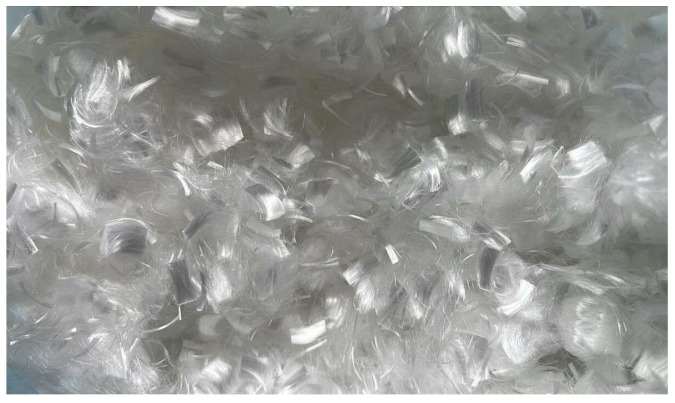
Polypropylene fiber waste.

Polypropylene fiber waste (PFW) consists of post-production or post-consumer remnants of polypropylene fiber remnants (Figure 2), fibers commonly used in construction, textiles, and packaging. Incorporating these fibers into construction materials, particularly concrete or mortar, can enhance mechanical properties such as tensile strength, crack resistance, and toughness, while also promoting sustainable waste management by diverting plastic waste from landfills.

Due to their chemical inertness, low density, and ease of dispersibility, polypropylene fibers represent a versatile and sustainable reinforcement material suitable for both conventional and advanced composites, a conclusion supported by the properties presented in Table 3.

### 2.4. Nanoparticulate in Civil Engineering Materials

Nanoparticles have increasingly attracted attention in the field of construction materials [16,17,19,20,21,22,23,24] due to their unique physicochemical properties and nanoscale dimensions. Owing to their nanometric size, these particles are influenced by quantum physics, which gives rise to unusual mechanical, optical, and chemical behaviors not observed in bulk forms. When incorporated into concrete or mortar, cement, coatings, or composites, nanoparticles such as titanium dioxide (TiO_2_), silica (SiO_2_), or carbon-based nanomaterials can enhance mechanical strength, durability, crack resistance, and impart additional functionalities like self-cleaning or antimicrobial activity. The integration of nanoparticles represents a promising avenue for developing more sustainable, high-performance, and multifunctional construction materials.

#### 2.4.1. Multi-Walled Carbon Nanotubes

**Figure 3:**
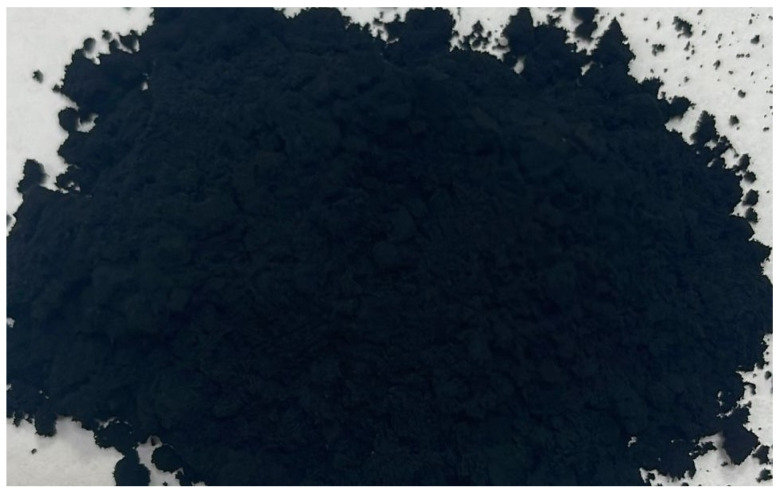
Multi-walled carbon nanotube.

Multi-Walled Carbon Nanotubes (MWCNTs), procured from Nanoshel LLC (Wilm1ington, DE, USA) [33] consist of multiple concentric graphene cylinders [12,13] nested within one another (Figure 3), offering unique mechanical, electrical, and thermal properties. Their high flexural strength, large surface area, and exceptional conductivity make them highly suitable for reinforcing construction materials, such as cement and concrete or mortar, improving durability, crack resistance, and overall structural performance. Additionally, MWCNTs can impart multifunctional characteristics, including enhanced thermal stability, electrical conductivity, and potential self-sensing capabilities, opening new avenues for advanced and sustainable construction applications.

#### 2.4.2. Nano-Silica Powder

**Figure 4:**
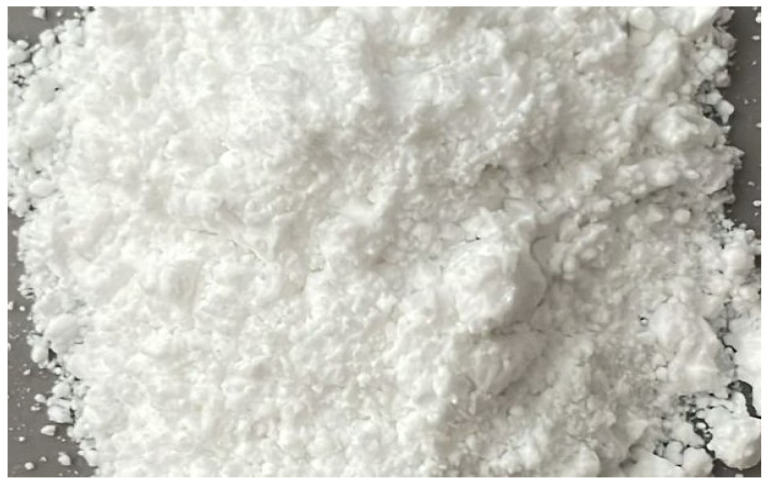
Nano-Silica Powder.

Nano-Silica Powder (NSP) surced from Nanoshel LLC (Wilmington, DE, USA) [33], is an ultrafine form of silicon dioxide [23,34] with particle sizes typically below 100 nm (Figure 4), characterized by a high specific surface area and enhanced reactivity compared to conventional silica. When incorporated into construction materials such as cement, concrete or mortar, NSP acts as a pozzolanic material, promoting the formation of additional calcium–silicate–hydrate (C–S–H) gel, which improves compressive strength, durability, and resistance to microcracking.

Its nanoscale dimensions also enable it to fill pores and refine the microstructure, contributing to denser, more homogeneous materials. Furthermore, NSP can enhance thermal stability and chemical resistance, making it a highly promising additive for advanced and sustainable construction applications.

#### 2.4.3. Demolition Waste Aggregates

**Figure 5:**
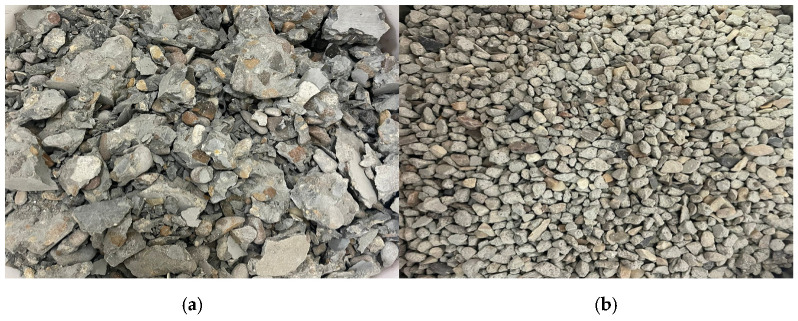
Demolition concrete waste aggregates: (**a**) crushed concrete (**b**) big sieve.

Demolition waste aggregates (DWA) are materials recovered from the deconstruction of existing structures, primarily consisting of concrete, masonry, and other construction debris. Among these, concrete waste represents a significant fraction, which can be crushed and processed to produce recycled aggregates [2,3] suitable for new concrete production. Utilizing demolition concrete waste aggregates (DCWA) (Figure 5) not only conserves natural resources but also reduces environmental impact by minimizing construction and demolition waste disposal. Furthermore, careful selection, processing, and quality control of recycled concrete aggregates can ensure the mechanical performance, durability, and compatibility comparable to conventional natural aggregates, making them a sustainable alternative in modern construction practices.

**Figure 6:**
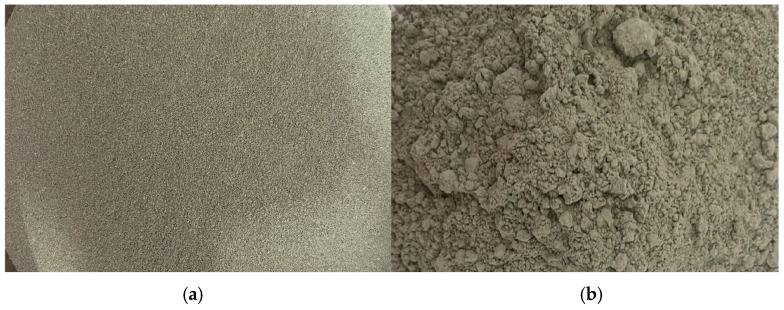
Recycled waste concrete aggregate: (**a**) small sort (**b**) fine part.

Recycled Concrete Fines (RCF) are a sustainable product developed by Holcim [31], obtained from the processing of crushed concrete and recycled construction materials (Figure 6b). They are used as a secondary raw material in cement and concrete production, helping to reduce the consumption of natural aggregates and support circular construction practices.

Principal characteristics of RCF:

- Contain ≥ 90% concrete, mortar, or natural stone.
- Include ≤ 10% clay masonry materials (bricks, tiles).
- Contain ≤ 1% bituminous materials.
- Floating materials ≤ 2 cm^3^/kg.
- Glass and other impurities ≤ 1%.
- TOC (Total Organic Carbon): ≤0.8% by mass.
- Sulfate content (as SO_3_): ≤2.0% by mass.
- Clay content: ≤1.20 g/100 g (tested with methylene blue method).
- Typical fineness: around 5000 cm^2^/g (Blaine specific surface).
- Mainly non-reactive, with minor residual hydraulic or pozzolanic activity.

#### 2.4.4. Taguchi Design of Experiments Methods

Taguchi Design of Experiments (DOE) methods [35,36] are a robust statistical approach developed to optimize processes and improve product quality with a minimal number of experiments [27,28,29,30]. By systematically studying the influence of multiple factors and their interactions, Taguchi methods aim to identify optimal conditions that minimize variability and enhance performance. Widely applied in engineering, materials science, and manufacturing, this methodology uses orthogonal arrays, signal-to-noise ratios, and robust parameter design to achieve efficient, cost-effective, and high-quality outcomes.

The Taguchi DOE approach offers an efficient strategy to optimize mortar reinforced with MWCNTs, NSP, and DWA. This method allows rapid identification of critical factors, resulting in a mortar with improved strength, durability, and sustainability.

#### 2.4.5. Ishikawa Diagram

**Figure 7:**
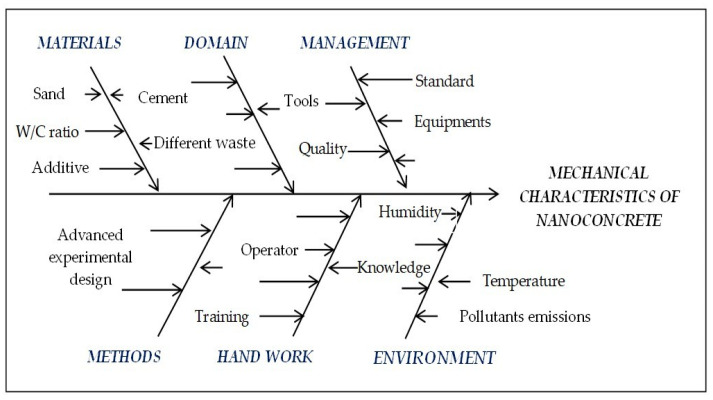
Ishikawa diagram for nanomortar or nanoconcrete.

To systematically identify and analyze the potential factors influencing the performance and durability of mortar mixes with nanomaterials, an Ishikawa (cause–effect or fishbone) diagram [27,28,29] (Figure 7) was employed. This method provides a structured visualization of possible sources of variation, categorizing them into management, materials, mixing method, environment, measurements, and human resources. By mapping these causes, the diagram helps to highlight the most critical factors (cement, water, sand, w/c ratio, additive, tools, equipment, operators, training, waste, etc.) that can lead to deficiencies such as increased porosity, reduced mechanical strength, or poor durability under freeze–thaw cycles and scouring (Figure 7). Importantly, the integration of nanomaterials (such as silica nanoparticles and carbon nanotubes) into the diagram emphasizes both their potential to enhance microstructural properties and the specific challenges associated with their dispersion, compatibility, and dosage optimization. In line with this analysis, the Taguchi experimental design was applied to systematically evaluate these factors and to determine their relative influence on the overall performance of the mortar.

#### 2.4.6. Drying of Mortar Samples Under Laboratory and Optimal Conditions

In order to ensure the accuracy and reproducibility of experimental results, mortar samples must be subjected to controlled curing and drying regimes. Laboratory drying reflects ambient conditions, but when higher precision and comparability are required, standardized procedures are recommended.

**Figure 8:**
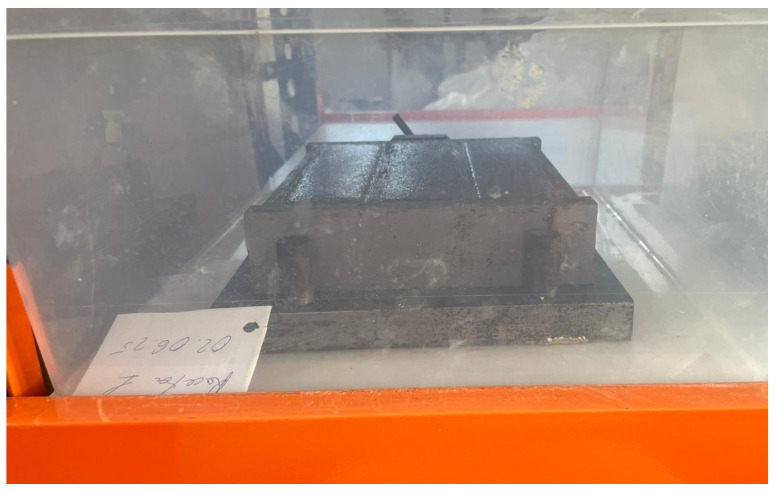
Drying of mortar samples drying process.

In this study, the samples were kept under optimal conditions in accordance with standard requirements [32], ensuring a controlled temperature and humidity regime (Figure 8). Specifically, the samples were cured at a relative humidity (RH) of ≥95% up to 28 days to promote proper hydration, and subsequently conditioned at approximately 65% RH until 56 days to achieve stable microstructural development prior to testing. Such an approach minimizes external variability, facilitates the proper development of mechanical properties, and provides a reliable basis for evaluating mortar performance in subsequent tests.

#### 2.4.7. Superplasticizer Additive in Mortar Mixes

**Figure 9:**
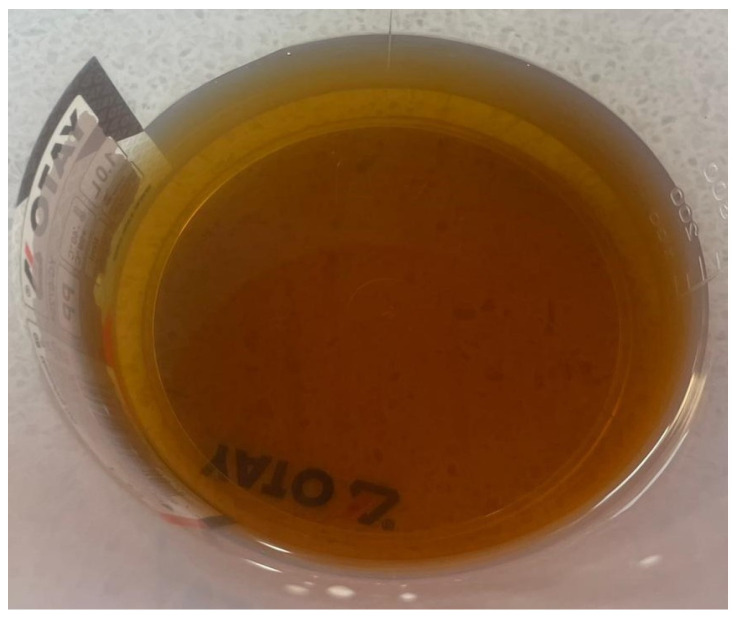
Superplasticizer additive—Sika Plastiment BV-101 N [37].

In this study, a superplasticizer (Figure 9) an essential component in high-strength self-compacting concrete (SCC) due to its ability to significantly reduce the water-to-cement ratio, was incorporated at the dosage established through Taguchi experimental design. Beyond improving workability and flowability, the use of superplasticizers in mortar mixtures enhances strength development, minimizes segregation and bleeding, improves durability by reducing porosity, and contributes to a more uniform microstructure.

For these specimens, Sika Plastiment BV-101 N [37] sourced from Sika, Bucharest Romania, was used as a water-reducing admixture to improve workability, reduce water content, and enhance cement dispersion. When incorporated into the mix, it reduces internal friction between cement particles and aggregates, promoting a more uniform and cohesive mortar. This results in improved workability at the same water-to-cement ratio, decreased water demand for a given consistency, and a homogeneous mix with minimal tendencies for segregation. Overall, the use of this additive ensures better fresh-state properties while supporting the development of high-performance mechanical and durability characteristics in the hardened material.

#### 2.4.8. Experimental Method

The experimental design incorporated seven key factors: *X*_1_ (silica nanoparticles), *X*_2_ (carbon nanotubes), *X*_3_ (aggregates derived from demolition waste), *X*_4_ (additive superplasticizer), *X*_5_ (cement), *X*_6_ (water), and *X*_7_ (polypropylene fibers recovered from waste). Each parameter was investigated at two distinct levels, as shown in Table 4. This approach not only ensures a systematic evaluation of multiple variables but also highlights the potential of integrating nanomaterials and recycled components in sustainable mortar design.

A controlled experimental program was implemented using the Taguchi L8 orthogonal array (Table 5) to ensure an efficient and statistically balanced exploration of eight mortar mixtures. The exact mix proportions, specimen identification codes and measured properties for all experimental runs are provided in Table 5.

**Figure 10:**
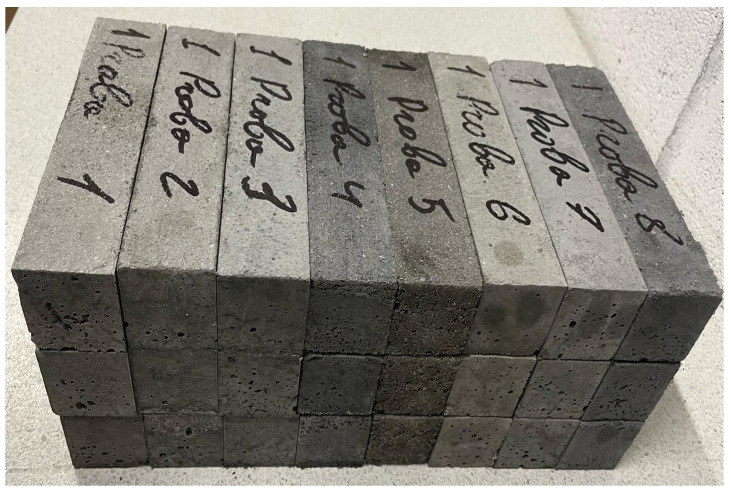
Experimental samples for nanomortar.

A preliminary dry-mixing stage of 180 s was implemented to promote the uniform homogenization of all solid constituents (cement, sand, recycled concrete waste, fibers, and nanoparticles). Although such a step is not explicitly specified in EN 196-1 [32], its inclusion was deemed necessary to enhance the dispersion efficiency of the solid phases prior to the introduction of water. For each experimental condition defined in the Taguchi L8 design matrix (Table 5), three replicate specimens (40 × 40 × 160 mm) were prepared and tested (Figure 10) in accordance with the relevant European standard—EN 196-1 [32], for compressive strength or flexural strength determination. The results corresponding to the two mechanical characteristics under investigation, flexural strength *Y*_1_ and compressive strength *Y*_2_ were subsequently reported as the mean values of the three independent measurements, in order to minimize random variability and ensure statistical reliability of the data.

## 3. Results

### Mechanical Characteristic Investigation

**Figure 11:**
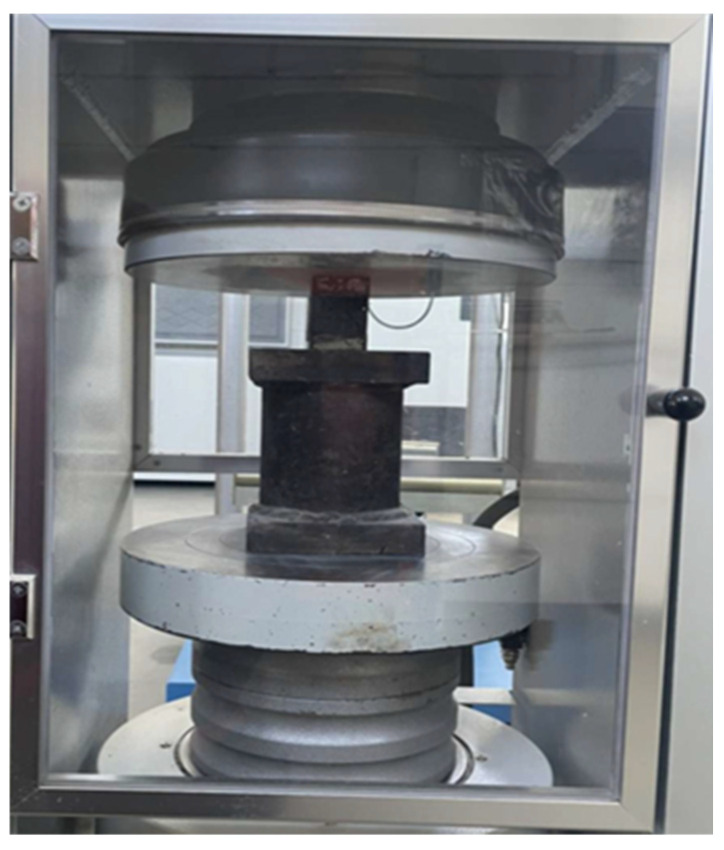
Compression test machine.

**Figure 12:**
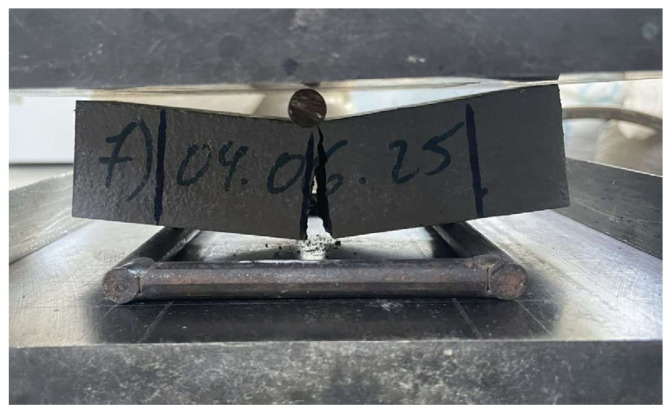
Flexural test machine.

The mechanical tests were performed after both 28 and 56 days of curing; however, only the results at 56 days are presented. This choice was made for two main reasons: (1) the scientific literature indicates that the effects of nanoparticles tend to stabilize over time, so later-age data are more representative, and (2) 56-day measurements provide a more reliable assessment of long-term mechanical behavior and allowed for the use of the most accurate testing setups. Two different presses were employed: a hydraulic compression press (Figure 11) for the compressive strength tests and a dedicated bending (flexural) testing press (Figure 12) for the flexural strength tests.

These machines differ in design and measurement accuracy, and each test was carried out on the press best suited and selected according to its accuracy to ensure the most precise results.

In this research, eight mortar formulations were designed and analyzed using the Taguchi methodology, with an L8 orthogonal array (Table 6). The table also includes the results of the experimental tests for *Y*_1_ (flexural strength) and *Y*_2_ (compressive strength), carried out in accordance with European standard [32], allowing a systematic assessment of the influence of the selected factors and their interactions. This approach provides a solid framework for optimizing mortar performance in terms of workability, mechanical strength, and durability under freeze–thaw and scouring conditions.

## 4. Discussions

In order to evaluate the influence of the experimental factors on the mechanical properties of ecological mortar, a statistical analysis was carried out based on the experimental design results. The aim of this analysis is to establish quantitative models linking the process parameters (*X*_1_, *X*_2_, …, *X*_7_) to the two main responses: Flexural Strength (*Y*_1_) and Compressive Strength (*Y*_2_). Using a linear model approach, the main effects of each factor were estimated from the measured responses, allowing identification of the most significant parameters and their influence trends. Let US PRESENT The model for the two objective responses, *Y*_1_: Flexural Strength and *Y*_2_: Compressive Strength

*Y*_1_ = *Y*_10_ + *a*_11_*X*_1_ + *a*_12_*X*_2_ + *a*_13_*X*_3_ + ⋯ + *a*_17_*X*_7_, (1)

*Y*_2_ = *Y*_20_ + *a*_21_*X*_1_ + *a*_22_*X*_2_ + *a*_23_*X*_3_ + ⋯ + *a*_27_*X*_7_. (2)

*Y*_10_ (respectively, *Y*_20_) represent the average of response *Y*_1_ (respectively, *Y*_2_ response). From the measurements obtained from the design plan (Table 6) and using some elementary calculus, we can estimate the effect (*a_ij_*) of each parameter and for both responses, Table 7 and Table 8.

**Figure 13:**
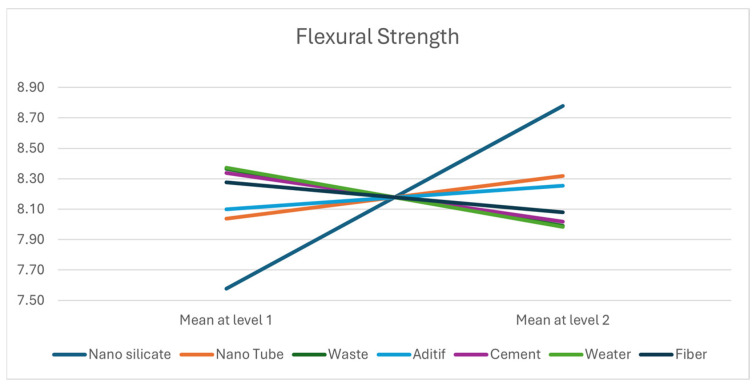
Mean response level for Flexural Strength.

**Figure 14:**
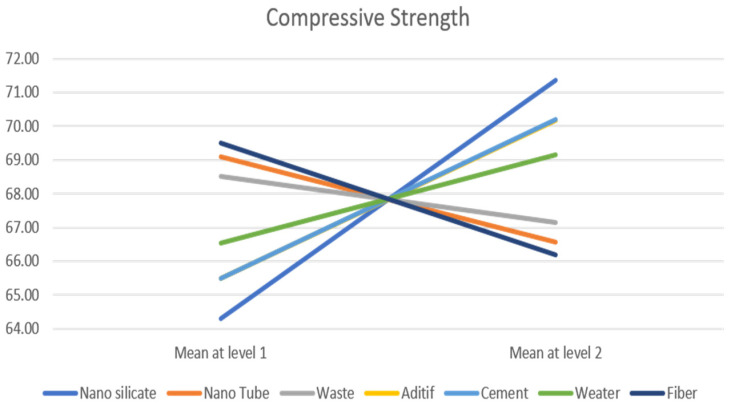
Mean response level for Compressive Strength.

We note that the parameters *X*_1_, *X*_6_ and *X*_3_ have significant effects on the response *Y*_1_ Flexural Strength and that parameters *X*_1_, *X*_5_ and *X*_4_ have an effect on the response *Y*_2_ Compressive Strength. Figure 13 and Figure 14, respectively, give the mean responses for each level of each parameter on the response *Y*_1_ (respectively, *Y*_2_).

On the other hand, if we consider, for example, the parameter Silica nanoparticles at its level 1 (2 g) the Flexural Strength response decreases by 0.6 with respect to mean value response and increases by 0.6 on the level 2 (4 g). Noting that the same parameter has an effect of 3.54 on the Compressive Strength response.

To determine the parameter with the greatest effect or order of parameters that has the greatest effect on both responses, we use the Pareto approach by calculating the contribution for each parameter on the responses. We use the equation below:

(3) $${contr}_{1j}=\frac{a_{1j}^{2}}{\sum_{j=1}^{7}a_{1j}^{2}}$$

(4) $${contr}_{2j}=\frac{a_{2j}^{2}}{\sum_{j=1}^{7}a_{2j}^{2}}$$

The tables (Table 9 and Table 10) highlight the partial and cumulative contributions of each parameter to the analyzed response.

**Figure 15:**
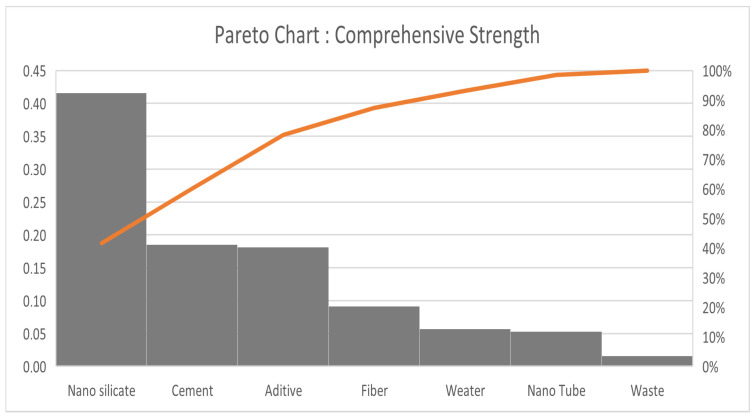
Pareto chart for Compressive Strength.

**Figure 16:**
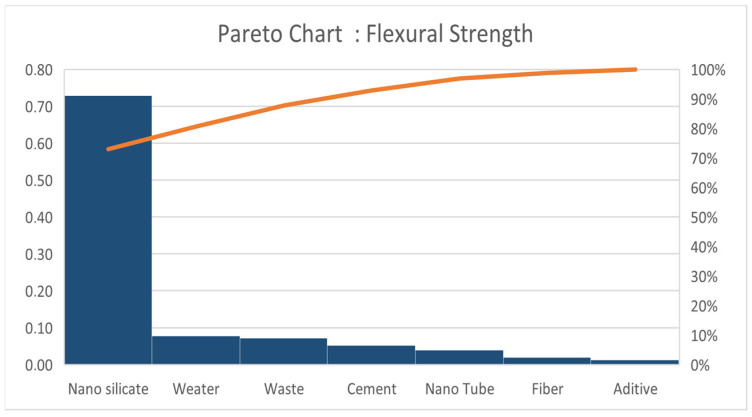
Pareto chart for Flexural Strength.

The results and Pareto chart (Figure 15 and Figure 16) clearly show that the first three parameters (nano-silica, cement and additive) contribute more than 78% (Table 9) to the construction of response *Y*_2_ (Figure 15), while for response *Y*_1_ (Figure 16), only two parameters, Nano Silicate and Water, for a contribution of 81% (Table 10).

The Analysis of Variance (ANOVA) [38,39] was employed as a complementary approach to the Pareto analysis in order to statistically validate and quantify the relative influence of each factor on the studied responses. While the Pareto chart provides a visual representation of the factors’ effects, the ANOVA method (Table 11) allows for a rigorous assessment of their statistical significance and contribution to the total variance. Regarding the *Y*_2_ response, similar conclusions can be drawn. The factors exerting the greatest influence, in decreasing order of effect according to the ANOVA results (Table 11), are *X*_1_, *X*_5_, *X*_4_, *X*_7_, *X*_6_, and *X*_2_. Once again, to adequately model the *Y*_2_ response, the first three factors collectively account approximately 78% of the total contribution. For the Compressive Strength (Response—*Y*_2_), the most influential parameters are Nano Silicate, Cement, and Additive, which together account for more than 78% of the total contribution to the response. The dominant effect of Nano Silicate can be attributed to its high surface reactivity and its ability to accelerate the hydration and pozzolanic reactions, leading to the formation of additional calcium silicate hydrate (C–S–H) phases and a denser microstructure. 

Cement plays its essential role as the primary binder, determining the bulk strength of the matrix, while the additive (likely a superplasticizer) improves the homogeneity and particle dispersion, indirectly enhancing hydration efficiency and the compressive response of the composite material.

Using the ANOVA (Table 12) approach, similar conclusions were obtained. For the response variable *Y*_1_ (Flexural Strength), the nano-silicate factor (*X*_1_) was found to be highly significant (*p* = 0.00279), while the other factors showed no statistically significant effects. This parameter alone accounts for approximately 73% of the variation in the *Y*_1_ response model. To increase the explanatory power beyond 80%, it would be necessary to include the water parameter (*X*_6_), although its associated *p*-value remains relatively high (0.2683), indicating limited statistical significance.

According to the Pareto diagram (Figure 16), only nano-silica and the water-to-cement (w/c) ratio exhibit a significant influence on the flexural strength (response—*Y*_1_), accounting for approximately 81% of the total effect. This indicates that the tensile behavior is primarily governed by the interaction between nanoparticle dispersion and water availability during hydration. A properly balanced w/c ratio ensures effective dispersion of nano-silica, minimizing microcrack formation and improving the bond at the paste–aggregate interface. The observed synergy between nano-silica and w/c ratio thus enhances the tensile resistance and overall ductility of the material.

Overall, these findings demonstrate that optimizing nano-silica dosage, cement proportion, and w/c ratio is essential for improving both compressive and tensile performance, providing a robust pathway for mix design optimization and enhanced mechanical behavior of nano-modified cementitious composites.

## 5. Conclusions

The research underscored the importance of employing modern experimental design methodologies to systematically analyze the factors influencing the mechanical behavior of concrete incorporating nanomaterials and recycled aggregates. The implementation of the Taguchi design of experiments allowed for the simultaneous evaluation of seven influencing parameters through only eight experimental mixtures, thereby achieving a significant optimization of time, cost, and experimental resources. This modern approach provided a statistically robust framework for understanding complex parameter interactions and for identifying the optimal composition configurations.

Although the combined incorporation of nanosilica powder and multiwalled carbon nanotubes has been widely reported to exhibit synergistic effects in enhancing the mechanical strength and durability of cement-based composites, in the present study such synergy was not conclusively observed. This outcome may be attributed to the specific proportions employed and the dispersion method applied, which relied primarily on mechanical mixing. The limited dispersion efficiency could have hindered the uniform distribution and effective interaction of the nano-additives within the cementitious matrix, thereby constraining the expected enhancement in microstructural densification and interfacial transition zone refinement. Consequently, while individual contributions of the nanomaterials were evident, their synergistic effect could not be clearly demonstrated under the adopted experimental conditions. However, a clear synergistic interaction can be highlighted between nanosilica, cement, the chemical admixture, and fibers, which contributed to the optimization of the hydration processes and to the local improvement of the microstructure, as well as to the increase in the compressive strength. The lack of direct interaction between the two types of nanoparticles could be attributed to factors such as inadequate dosage, the cumulative influence of other parameters, or the delayed manifestation of quantum bonding effects, which may become more pronounced during the later stages of the hardening process.

Nevertheless, the composites exhibited a notably improved mechanical performance. For sample 6, the compressive strength increased by approximately 21%, accompanied by a 7.3% enhancement in flexural strength compared to the reference mix, whereas sample 8 demonstrated a 9.5% increase in compressive strength and a 21% improvement in flexural strength. These results clearly indicate a consistent strengthening effect associated with the combined action of nanosilica and fibrous reinforcement within the cementitious matrix. These enhancements are mainly associated with the presence of nanosilica, whose high surface energy and pozzolanic activity promote microstructural densification, refinement of the interfacial transition zone, and overall strength development. The contribution of multiwalled carbon nanotubes, though less dominant, remains relevant, particularly in improving crack control and local stress redistribution. The overall improvement therefore reflects the beneficial but non-synergistic influence of both nanomaterials, operating through distinct mechanisms at the nanoscale, where surface reactivity, particle size, and quantum level interactions play a significant role in strengthening the cementitious matrix.

Furthermore, the integration of these advanced nanomaterials with recycled demolition waste aggregates highlights an emerging and sustainable pathway for the concrete industry, aiming to minimize environmental impact while preserving or even improving material performance. Overall, the combined application of nanotechnology and modern Taguchi-based experimental optimization demonstrates substantial potential for developing next-generation sustainable, high-performance cementitious composites, bridging the gap between materials engineering and nano-scale physics. Future research will focus on extending the experimental program to include microstructural analyses (SEM, XRD, FTIR, MIP) and durability tests such as resistance to freeze–thaw cycles and water permeability. These investigations will help validate the current findings and provide a deeper understanding of the material’s long-term behavior.

## Figures and Tables

**Table 1:** Properties of Portland cement.

Nr.	Value	Unit
1	Specific Gravity	3.15
2	Consistency (%)	32
3	Initial Setting time (min)	30
4	Final Setting time (min)	600
5	Fineness modulus (%)	3.60
*Fine aggregate characteristics*		
1	Specific Gravity	2.8
2	Fineness modulus (%)	3.70
3	Bulk density (g/cc)	1.843

**Table 2:** Sand granular characteristics.

Nr.	Sand Particle Dimension (mm)	Quantity (g)
1	0.06–0.16	150
2	0.16–0.50	300
3	0.50–1.00	450
4	1.00–2.00	450

**Table 3:** Fiber characteristics.

Nr.	Name	Value	Unit
1	Fiber length	6–18	mm
2	Fiber diameter	20–30	µm
3	Fiber melting point	>165	°C
4	Fiber density	0.91	kg/L
5	Fiber consumption	600	g/m^3^

**Table 4:** Parameter level.

Parameters							
Level	*X*_1_	*X*_2_	*X*_3_	*X*_4_	*X*_5_	*X*_6_	*X*_7_
	(g)	(g)	(g)	(mL)	(g)	(mL)	(g)
Up	4	3	200	2.5	445	225	1.5
Down	2	1	100	1.5	370	187.5	0.5

**Table 5:** Mix proportions—Taguchi L8 design.

Parameters							
No.	*X*_1_	*X*_2_	*X*_3_	*X*_4_	*X*_5_	*X*_6_	*X*_7_
	(g)	(g)	(g)	(mL)	(g)	(mL)	(g)
1	2	1	100	1.5	370	187.5	0.5
2	2	1	100	2.5	445	225	1.5
3	2	3	200	1.5	370	187.5	1.5
4	2	3	200	2.5	445	225	
5	4	1	200	1.5	445	225	1.5
6	4	1	200	2.5	370	187.5	0.5
7	4	3	100	1.5	445	225	0.5
8	4	3	100	2.5	370	187.5	1.5

**Table 6:** Results of mixture proportions—Taguchi L8 design.

Parameters									
No.	*X*_1_ (g)	*X*_2_ (g)	*X*_3_ (g)	*X*_4_ (mL)	*X*_5_ (g)	*X*_6_ (mL)	*X*_7_ (g)	*Y*_1_ (N/mm^2^)	*Y*_2_ (N/mm^2^)
1	2	1	100	1.5	370	187.5	0.5	8.00	61.9
2	2	1	100	2.5	445	225	1.5	7.25	70.6
3	2	3	200	1.5	370	187.5	1.5	7.32	57.3
4	2	3	200	2.5	445	225	0.5	7.74	67.4
5	4	1	200	1.5	445	225	1.5	8.31	69.0
6	4	1	200	2.5	370	187.5	0.5	8.59	74.9
7	4	3	100	1.5	445	225	0.5	8.77	73.8
8	4	3	100	2.5	370	187.5	1.5	9.44	67.8

**Table 7:** Effect for (*Y*_1_) response—Flexural Strength.

Parameters							
Statistics	*X*_1_	*X*_2_	*X*_3_	*X*_4_	*X*_5_	*X*_6_	*X*_7_
Mean at level 1	7.58	8.04	8.37	8.1	8.34	8.37	8.28
Mean at level 2	8.78	8.32	7.99	8.26	8.02	7.98	8.08
Effect (*a_ij_*)	−0.6	−0.14	0.18	−0.07	0.16	0.19	0.09

**Table 8:** Effect for (*Y*_2_) response—compressive strength.

Parameters							
Statistics	*X*_1_	*X*_2_	*X*_3_	*X*_4_	*X*_5_	*X*_6_	*X*_7_
Mean at level 1	64.30	69.10	68.53	65.50	65.48	66.53	69.50
Mean at level 2	71.38	66.58	67.15	70.18	70.20	69.15	66.18
Effect (*a_ij_*)	−3.54	1.26	0.69	−2.34	−2.36	−1.31	1.66

**Table 9:** Contribution for Compressive Strength response.

Parameter	Contribution	Cumulate Contribution
Nano silicate	0.42	0.42
Cement	0.19	0.60
Additive	0.18	0.78
Fiber	0.09	0.87
Water	0.06	0.93
Nano Tube	0.05	0.98
Waste	0.02	1.00

**Table 10:** Contribution for Flexural Strength response.

Parameter	Contribution	Cumulate Contribution
Nano silicate	0.73	0.73
Water	0.08	0.81
Waste	0.07	0.88
Cement	0.05	0.93
Nano Tube	0.04	0.97
Fiber	0.02	0.99
Additive	0.01	1.00

**Table 11:** ANOVA table for Compressive Strength response.

Factor	SS	df	Variance	F	*p*-Value
Nano-silica	300.33375	1	300.33375	38.956481	0.0011781
Nano-tube	38.25375	1	38.25375	4.96191815	0.04061414
Waste	11.34375	1	11.34375	1.471405	0.2427297
Additive	131.13375	1	131.13375	17.0094418	0.00079509
Cement	133.95375	1	133.95375	17.3752258	0.00072493
Water	41.34375	1	41.34375	5.36272401	0.03416942
Fiber	66.33375	1	66.33375	8.60419274	0.00974336
*Y*_2_	123.351491	16	7.70946816	1	
Total	846.047741	23			

**Table 12:** ANOVA table for Flexural Strength response.

Nano Silicate	8.64	1	8.64	12.4502405	0.00279047
Nano Tube	0.4704	1	0.4704	0.67784643	0.4224318
Waste	0.84375	1	0.84375	1.2158438	0.28648772
Additive	0.14415	1	0.14415	0.20772016	0.65468487
Cement	0.6144	1	0.6144	0.88535043	0.36073062
Water	0.9126	1	0.9126	1.31505665	0,26834164
Fiber	0.22815	1	0.22815	0.32876416	0.5743591
*Y*_1_	11.1034	16	0.6939625	1	
Total	22.95685	23			

## Data Availability

The original contributions presented in this study are included in the article. Further inquiries can be directed to the corresponding authors.

## Abbreviations

The following important abbreviations are used in this manuscript:

MWCNTs	Multi-Walled Carbon Nanotubes
DoE	Design of Experiments
CNT	Carbon nanotubes
PFW	Polypropylene fiber waste
DWA	Demolition waste aggregates
DCWA	Demolition concrete waste aggregates
NSP	Nano-silica powder
SCC	Self-compacting concrete
SEM	Scanning Electron Microscopy
XRD	X-ray Diffraction
SC-XRD	Single-Crystal X-Ray Diffraction
FTIR	Fourier Transform Infrared Spectroscopy
MIP	Mercury Intrusion Porosimetry
EN	European Norme
CEM	Cement
